# Lifestyle Factors and Quality of Life among Patients with Chronic Diseases at the Primary Healthcare Centers in Riyadh, Saudi Arabia

**DOI:** 10.4314/ejhs.v34i6.8

**Published:** 2024-11

**Authors:** Reem S Alanezi, Waad A Alasmari, Basma S Almutairi, Zainab A Albalawi, Wedad A Alasmari, Qassem M Alotiby, Afaf M Alosaimi, Amal A Alahmari, Ahlam S Alharthi, Safa M Faizo

**Affiliations:** 1 Department of Nursing, Al Narjis Health Center, Riyadh, Saudi Arabia; 2 Department of Nursing, Riyadh Military Hospital, Riyadh, Saudi Arabia; 3 Department of Nursing Management, Riyadh Third Health Cluster, Riyadh, Saudi Arabia; 4 Blood Bank Center, Riyadh, Saudi Arabia; 5 Department of Nursing, King Saud Medical City (KSMC), Riyadh, Saudi Arabia

**Keywords:** Chronic diseases, Lifestyle factors, Riyadh, Primary health care centers, Quality of life, Saudi Arabia

## Abstract

**Background:**

The current study was conducted to assess the common lifestyle risk factors affecting the quality of life (QoL) among patients with chronic diseases.

**Methods:**

This cross-sectional study was conducted using a valid structured questionnaire among 734 patients with chronic diseases. Patients were randomly selected from five primary health care centers in Riyadh of Saudi Arabia during January to February 2024. The study survey included a checklist of socio-demographic and lifestyle variables; the Arabic short version of the World Health Organization QoL questionnaire was used. The SPSS (version 24) was used for data analysis.

**Results:**

A total of 734 patients (14.7% males and 85.3% females) were included. The mean age of the patients was 48.54±19 years. The total QoL and its four domains mean scores were relatively high, with no statistically significant differences were found between males and females. Statistically significant reductions in the positive (good) QoL were found in patients with obesity; patients who using butter and animal fat in cooking; and patients who eating meals out > 3 times per week (OR 0.64 CI 95% (0.325-0.891)), (OR 0.21 CI 95% (0.031-0.754)), and (OR 0.42 CI 95% (0.112-0.851)) respectively.

**Conclusion:**

The current study shows high level (69.3%) of good QoL among patients with chronic diseases at the primary healthcare centers in Riyadh of Saudi Arabia. Furthermore, obesity, poor cooking practices, and eating meals outside-home are the main unhealthy lifestyle factors that impaired the level of the QoL among the studied population.

## Introduction

Chronic diseases, also known as Non-communicable diseases (NCDs) are identified as a major public health concern worldwide, and contributor to a large burden of diseases in high income countries, and increasing rapidly in low- and middle-income countries (1, 2). The World Health Organization (WHO) estimated that the four main NCDs including cardiovascular diseases, cancer, diabetes and chronic respiratory diseases were responsible for 41 million deaths yearly and account for 71% of all deaths worldwide (3).

In developing countries, socioeconomic growth, fast urbanization, and epidemiological change have contributed to an upward trend in NCDs (4). Saudi Arabia has one of the highest rates of NCDs globally and the highest in the Arabian Gulf (5,6). Like most countries, the NCDs burden is a public health issue, resulting in significant mortality and morbidity; NCDs claim around 83,100 deaths every year, accounting for 73% of all deaths in the Kingdom (7). In addition to causing premature mortality, chronic diseases also hurt the economic well-being of individuals, households, and the community at large (8). In Saudi Arabia, NCDs are responsible for almost 78% of annual deaths, with cardiovascular disorders, cancers, chronic respiratory diseases, and diabetes being the most prevalent (8,9).

Quality of life (QoL) is considered one of the measures of health outcomes. In addition, there has been great concern about the role of lifestyle factors in preventing of chronic diseases and improving the QoL (10). Patients diagnosed with chronic diseases are a vulnerable group for a poor QoL (11); however, limited research studies have assessed the common lifestyle risk factors and QoL among them. Therefore, the current study was conducted to assess the common lifestyle risk factors affecting the QoL among patients with chronic diseases at the primary health care centers in Riyadh of Saudi Arabia.

## Methods

**Study design and period**: This cross-sectional study was conducted among a representative sample of patients with chronic diseases during January to February 2024. It was conducted to assess the common lifestyle risk factors affecting the QoL among patients with chronic diseases at the primary health care centers in Riyadh of Saudi Arabia.

**Study setting**: The current study was conducted at five (level IV/centers with a high degree of specialization) governmental primary health care centers in Riyadh of Saudi Arabia during January to February 2024.

**Study participants and sampling technique**: A representative sample of 734 patients with chronic diseases, aged ≥ 18 years, both genders, was calculated using the Charan and Biswas formula (12). In Riyadh of Saudi Arabia, only five (level IV) governmental primary health care centers were accredited by the Saudi Central Board for Accreditation of Healthcare Institutions (CBAHI) (13). From these centers (Al Amir Sultan Ibn Abdul Aziz center, Atiqa center, District Al-Shifa Al-Awal center, Al Rawda I center, and AlKhaleej II center) patients with chronic diseases were randomly selected based on the population density in each of them.

**Eligibility criteria (inclusion and exclusion criteria)**: Patients who diagnosed with any type of chronic diseases by the physicians, and had medical records for follow up at the primary health care centers were included in the study. In contrary, patients with serious condition such as acute myocardial infarction, end stage renal disease, pregnant, and lactating women were excluded.

### Data collection

**Study questionnaire**: In the current study, data were collected using face-to-face, pre-tested and validated Arabic-structured questionnaire. The study survey included a checklist of sociodemographic data, known lifestyle factors and physical activity variables was adopted based on the World Health Organization (WHO) STEP-wise survey (14).

**Anthropometric measurements**: In addition, height (cm) and weight (kg) were measured in all patients using a standard method (15); the body mass index (BMI) was calculated by dividing weight in kilograms by the square of height in meters. BMI was classified into normal weight (18.5 to < 25 kg/m^2^), overweight (25 to < 30 kg/m^2^) and obesity (≥ 30 kg/m^2^) according to the WHO classification (16,17).

**QoL questionnaire**: A validated, Arabic short version of the WHO QoL questionnaire (WHOQOL-BREF) was used, which consist of twenty-six questions, and four domains as follow: physical domain (7 items), psychological domain (8 items), social domain (3 items), and environmental domain (8 items). In the current study, positive QoL includes those subjects whom domain score exceeds 65%; this cut-off point was based on a previous study (18).

**Pilot study**: The questionnaire was piloted among twenty of the eligible patients, to ensure the survey's acceptance and consistency. The results of the pilot study showed a good overall Cronbach's alphas of 0.84.

**Ethical issues**: The study protocol was approved by the Ethics Committee of Al Riyadh Third Health Cluster (No.: THC/HRC/33/2023). In addition, informed consent was obtained from each participant. Additionally, no monetary rewards were given for completing the questionnaire.

**Statistical analysis**: The Statistical Package for Social Science (SPSS) version 24 was used for data analysis. Descriptive statistics described the characteristics of the study participants. Frequencies and percentages were used to describe categorical variables, whereas mean values and standard deviations (SDs) were used to represent continuous variables. The differences between means were tested by using independent sample t test. P value less than 0.05 was considered as statistically significant. Additionally, multivariable logistic regression analyses were performed to examine the association of the QoL and its domains with the studied lifestyle risk factors, while controlling for possible confounding factors.

## Results

A total of 734 patients with chronic diseases (14.7% males and 85.3% females) were included in the final analysis. The mean age of the patients was 48.54±19 years. The majority of the patients were married, 55.5% had high education (high school and university), 64.2% had large family size (five or more), and most of them 84.8% had low monthly income (0 to 15299 Riyal Saudi). In addition, about 32.6% of the patients had chronic diseases (cardiovascular diseases, cancer, diabetes and chronic respiratory diseases) related to their lifestyle risk factors, and 38.5% of them had chronic diseases since more than ten years as shown in Table 1.

**Table 1:** Characteristics of the study participants **(N = 734)**

Variables		No. (%)
**Age (years)**	Mean±SD	48.54±19
	18 to 64 years	642 (87.5)
	More than 64 years	92.0 (12.5)
**Gender**	Male	108 (14.7)
	Female	626 (85.3)
**Marital status**	Married	709 (96.6)
	Unmarried	25.0 (3.4)
**Educational level**	Low education	327 (44.5)
	High education	407 (55.5)
**Family size**	Less than five	263 (35.8)
	Five or more	471 (64.2)
**Monthly income in Riyal Saudi**	0 to 15299	622 (84.8)
	15300 or more	112 (15.2)
**Chronic diseases duration (years)**	Less than five	186 (25.4)
	Five to ten	265 (36.1)
	More than ten	283 (38.5)
**Lifestyle medical related problems**	Yes	239 (32.6)
	No	495 (67.4)

Data are expressed as means ± SD for continuous variables and as percentage for categorical variables. Low education: Illiterate, elementary and intermediate; High education: High school and university

Table 2 show the mean QoL scores of the study participants by sex. The results revealed that the total QoL score was slightly higher in female patients when compared to male patients (279.5±48.7 vs. 275.7±56.3). In addition, the mean physical, psychological, social, and environmental QoL scores seem to be higher in male patients when compared to female patients. Furthermore, no statistically significant differences were found between males and females (p-value > 0.05 for all).

**Table 2:** Quality of life scores of the study participants by sex

Variables	Male (n=108) Mean±SD	Female (n=626) Mean±SD	P Value
Total quality of life scores	275.7±56.3	279.5±48.7	0.238
Physical quality of life scores	79.3±12.1	78.8±11.2	0.354
Psychological quality of life scores	71.4±10.6	70.1±11.0	0.521
Social quality of life scores	73.6±14.1	72.6±15.4	0.872
Environmental quality of life scores	68.1±10.2	67.8±10.4	0.650

Data are expressed as means ± SD for continuous variables. The differences between means were tested by using independent sample t test. P value less than 0.05 was considered as statistically significant. SD, stander deviation.

The association between lifestyle variables with positive QoL among patients with chronic diseases is shown in Table 3. The current study show high level (69.3%) of good total QoL, among patients with chronic diseases. After adjustment of potential confounding variables. The results revealed that statistically significant reductions in the positive QoL were found in patients with obesity (BMI ≥ 30 kg/m^2^); patients who using butter and animal fat in cooking; and patients who eating meals out > 3 times per week (OR 0.64 CI 95% (0.325-0.891)), (OR 0.21 CI 95% (0.031-0.754)), and (OR 0.42 CI 95% (0.112-0.851)) respectively, (P value <0.05 for all). No significant association was found between the positive QoL with other lifestyle variables.

**Table 3:** Lifestyle variables and their associations with positive quality of life among the study participants

Lifestyle variables		Positive quality of life	Negative quality of life	P Value	OR (95% CI)
		n=509 (69.3%)	n=225(30.7%)		
**Smoking**	Never	391 (76.8%)	181 (80.4%)	-	1.0 (Ref.)
	Current smokers	118 (23.2%)	44.0 (19.6%)	0.624	2.12 (0.631-4.13)
**Body mass index (kg/m^2^)**	< 25 (normal weight)	225 (44.2%)	64.0 (28.4%)	-	1.0 (Ref.)
	25 to < 30 (overweight)	194 (38.1%)	75.0 (33.4%)	0.735	0.51 (0.251-1.84)
	≥ 30 (obesity)	90.0 (17.6%)	86.0 (38.2%)	0.002	0.64 (0.325-0.891)
**Cooking oil**	Vegetable oil	494 (97.1%)	178 (79.1%)	-	1.0 (Ref.)
	Butter and animal fat	15.0 (2.9%)	47.0 (20.9%)	0.034	0.21 (0.031-0.754)
**Meal outside home/week**	≤ 3 times per week	238 (46.8%)	39.0 (17.3%)	-	1.0 (Ref.)
	> 3 times per week	271 (53.2%)	186 (82.7%)	0.029	0.42 (0.112-0.851)
**Total items of fruit intake/day**	≤ 3 servings	285 (56.0%)	120 (53.3%)	-	1.0 (Ref.)
	> 3 servings	224 (44.0%)	105 (46.7%)	0.541	3.1 (0.514-1.25)
**Total items of vegetables intake/day**	≤ 3 servings	215 (42.2%)	122 (54.2%)	-	1.0 (Ref.)
	> 3 servings	294 (57.8%)	103 (45.8%)	0.648	2.3 (0.921-5.34)
**Physical activity levels**	Low	160 (31.4%)	62.0 (27.6%)	-	1.0 (Ref.)
	Moderate	158 (31.0%)	120 (53.3%)	0.744	1.2 (0.214-0.797)
	High	191 (37.6%)	43.0 (19.1%)	0.543	1.5 (0.325-4.35)
**Sitting hours in front of TV, laptop, internet, etc./day**	≤ 3 hours	435 (85.5%	156 (69.3%)	-	1.0 (Ref.)
	> 3 hours	74.0 (14.5%)	69.0 (30.7%)	0.691	0.30 (0.205-0.803)

Positive quality of life includes those subjects whom domain score exceeds 65%. Reference group: Negative quality of life; OR: Odds Ratio; CI: Confidence Intervals. Adjusted for age, gender, marital status, educational level, family size, monthly income, chronic diseases duration, and lifestyle medical related problems.

Moreover, after adjustment of potential confounding variables. The results revealed that statistically significant reduction in the positive physical QoL was found in patients who eating meals out > 3 times per week (OR 0.71 CI 95% (0.516-.987)) (P value = 0.031). In addition, the positive physical QoL, was increased among patients with chronic diseases who eating >3 servings of fruit/day, and who eating >3 servings of vegetables/day, (OR 1.50 CI 95% (1.133-2.840)), and (OR 1.14 CI 95% (0.868-1.578)) respectively, (P-value <0.05 for all). No significant association was found between the positive physical and psychological QoL with other lifestyle variables as shown in Table 4.

**Table 4:** Lifestyle variables and their associations with positive physical and psychological domains of quality of life among the study participants

Lifestyle variables	Physical quality of life	P Value	OR (95% CI)	Psychological quality of life	P Value	OR (95% CI)
	Positive/Negative n=540/n=194			Positive/Negative n=507/n=227		
**Smoking**						
Never	482/90.0	-	1.0 (Ref.)	426/146	-	1.0 (Ref.)
Current smokers	58.0/104	0.641	0.36(0.089-1.820)	81.0/81.0	0.238	1.5(0.933-1.381)
**Body mass index (kg/m^2^)**						
< 25 (normal weight)	234/55.0	-	1.0 (Ref.)	203/86.0	-	1.0 (Ref.)
25 to < 30 (overweight)	183/86.0	0.702	2.4(1.248-4.150)	172/97.0	0.242	0.54(0.542-1.342)
≥ 30 (obesity)	123/53.0	0.149	0.79(0.279-2.386)	132/44.0	0.362	0.83(0.551-1.520)
**Cooking oil**						
Vegetable oil	518/154	-	1.0 (Ref.)	485/187	-	1.0 (Ref.)
Butter and animal fat	22.0/40.0	0.551	0.86 (0.657-1.031)	22.0/40.0	0.518	0.04(0.202-1.315)
**Meal outside home/week**						
≤ 3 times per week	254/23.0	-	1.0 (Ref.)	203/74.0	-	1.0 (Ref.)
> 3 times per week	286/171	0.031	0.71(0.516-.987)	304/203	0.927	1.03(0.936-1.261)
**Total items of fruit intake/day**						
≤ 3 servings	264/141	-	1.0 (Ref.)	284/121	-	1.0 (Ref.)
> 3 servings	276/53	0.001	1.50(1.133-2.840)	223/106	0.138	0.20(0.066-1.350)
**Total items of vegetables intake/day**						
≤ 3 servings	308/29.0	-	1.0 (Ref.)	325/12.0	-	1.0 (Ref.)
> 3 servings	232/165	0.044	1.14(0.868-1.578)	182/215	0.229	1.4(1.133-2.838)
**Physical activity levels**						
Low	183/39.0	-	1.0 (Ref.)	112/110	-	1.0 (Ref.)
Moderate	194/84.0	0.758	0.30 (0.256-1.750)	193/85.0	0.146	1.80(1.490-2.175)
High	163/71.0	0.347	1.15(0.890-1.452)	202/32.0	0.092	1.35(1.057-1.690)
**Sitting hours in front of TV, laptop, internet, etc./day**						
≤ 3 hours	453/138	-	1.0 (Ref.)	431/160	-	1.0 (Ref.)
> 3 hours	87.0/56.0	0.594	0.84(0.336-1.563)	76.0/67.0	0.052	1.32(1.141-2.615)

Positive physical and psychological aspects of quality of life include those subjects whom domain score exceeds 65%. Reference group: Negative quality of life; OR: Odds Ratio; CI: Confidence Intervals. Adjusted for age, gender, marital status, educational level, family size, monthly income, chronic diseases duration, and lifestyle medical related problems.

Additionally, after adjustment of potential confounding variables. The results revealed that statistically significant reductions in the positive social QoL was found in patients who currently smokers, patients with obesity, patients who eating meals out > 3 times per week, and patients who sitting > 3 hours in front of TV, laptop, internet, etc./day, (OR 0.63 CI 95% (0.013-0.305)), (OR 0.39 CI 95% (0.225-0.680)), (OR 0.43 CI 95% (0.197-0.942)) and (OR 0.16 CI 95% (0.050-0.557)) respectively, (P value <0.05 for all). Moreover, the positive social QoL, was increased among patients with chronic diseases who eating >3 servings of vegetables/day, (OR 1.27 CI 95% (0.799-2.034)) (P value = 0.004). No significant association was found between the positive social and environmental QoL with other lifestyle variables as shown in Table 5.

**Table 5:** Lifestyle variables and their associations with positive social and environmental domains of quality of life among the study participants

Lifestyle variables	Social quality of life	P Value	OR (95% CI)	Environmental quality of life	P Value	OR (95% CI)
	Positive/Negative n=625/n=109			Positive/Negative n=406/n=328		
**Smoking**						
Never	531/41.0	-	1.0 (Ref.)	346/226	-	1.0 (Ref.)
Current smokers	94.0/68.0	0.001	0.63(0.013-0.305)	60.0/102	0.542	0.99(0.794-1.249)
**Body mass index (kg/m^2^)**						
< 25 (normal weight)	256/33.0	-	1.0 (Ref.)	188/101	-	1.0 (Ref.)
25 to < 30 (overweight)	237/32.0	0.548	0.42(0.084-2.119)	129/140	0.375	0.46(0.285-0.763)
≥ 30 (obesity)	132/44.0	0.025	0.39 (0.225-0.680)	89.0/87.0	0.084	0.51(0.315-0.827)
**Cooking oil**						
Vegetable oil	603/69.0	-	1.0 (Ref.)	386/286	-	1.0 (Ref.)
Butter and animal fat	22.0/40.0	0.622	0.29(0.055-1.534)	20.0/42.0	0.298	0.51(0.320-0.841)
**Meal outside home/week**						
≤ 3 times per week	268/9.0	-	1.0 (Ref.)	178/99.0	-	1.0 (Ref.)
> 3 times per week	357/100	0.002	0.43(0.197-0.942)	228/229	0.355	0.95(0.816-1.123)
**Total items of fruit intake/day**						
≤ 3 servings	381/24.0	-	1.0 (Ref.)	209/196	-	1.0 (Ref.)
> 3 servings	244/85.0	0.362	0.49(0.281-0.885)	197/132	0.501	0.96(0.811-1.178)
**Total items of vegetables intake/day**						
≤ 3 servings	320/17.0	-	1.0 (Ref.)	247/90.0	-	1.0 (Ref.)
> 3 servings	305/92.0	0.004	1.27(0.799-2.034)	159/238	0.604	1.11(0.923-1.351)
**Physical activity levels**						
Low	203/19.0	-	1.0 (Ref.)	117/105	-	1.0 (Ref.)
Moderate	205/73.0	0.242	0.83(0.657-1.055)	129/149	0.186	0.70(0.179-3.485)
High	217/17.0	0.073	0.80(0.511-1.261)	160/74.0	0.671	0.36 (0.079-1.894)
**Sitting hours in front of TV, laptop, internet, etc./day**						
≤ 3 hours	518/73.0	-	1.0 (Ref.)	334/257	-	1.0 (Ref.)
> 3 hours	107/36.0	0.032	0.16(0.050-0.557)	72.0/71.0	0.616	0.51(0.142-1.342)

Positive social and environmental aspects of quality of life include those subjects whom domain score exceeds 65%. Reference group: Negative quality of life; OR: Odds Ratio; CI: Confidence Intervals. Adjusted for age, gender, marital status, educational level, family size, monthly income, chronic diseases duration, and lifestyle medical related problems.

## Discussion

The main findings of the current study indicated a high level (69.3%) of good (positive) total QoL among the studded population. A previous study found that the participants with any chronic disease demonstrated a poorer QoL compared with individuals who did not have a chronic disease diagnosis (19). When comparing the results of the current study with the previous studies (19, 20), different measures to assess the QoL were implemented; so, the ability to compare and discuss the QoL among those with chronic diseases was limited.

In the current study, obesity, poor cooking practices, and eating meals outside home are the main unhealthy lifestyle factors that impaired the level of the QoL among patients with chronic diseases. Obesity is known to influence health, in general, and it is considered to be an important risk factor for several diseases; as such, it has been reported to affect the health related QoL (20). In addition, butter and animal fat cooking is a risk factor for developing chronic diseases. The results of a recent study reported higher physical health related QoL scores among those reporting a daily intake of vegetable oils when compared with those reporting a daily intake of animal fat (21). The results of the current study support these findings. Moreover, greater food consumption away from home was associated with higher energy intake and poorer diet quality which associated with a high risk of chronic diseases and poor QoL (22). The results of the current study support these findings.

The results of the current study, revealed that the total QoL score was slightly higher in female patients when compared to male patients. In addition, the mean physical, psychological, social, and environmental QoL scores seem to be higher in male patients when compared to female patents; with no statistically significant differences were found between males and females. A previous study showed that women consistently reported poorer QoL than their male counterparts (23). Actually, more studies are recommended.

Moreover, the findings of the current study revealed that statistically significant reductions in the positive social QoL was found in patients who currently smokers, patients with obesity, patients who eating meals out > 3 times per week, and patients who sitting > 3 hours in front of TV, laptop, internet, etc./day. Findings from previous study suggest that for smokers, the average probability of having a higher quality of life was 11.65% lower than when they did not smoke (24). Additionally, a previous study showed that sitting in front of the TV, laptop, or internet for >3 hours per day was associated with reduction in the positive QoL of 60% (25). The results of the current study support these findings.

In Saudi Arabia, the ongoing health care system transformation, public-private partnerships, and the public sector's shift toward corporatization/privatization has a potential for improving the care of NCDs/lifestyle risk factors. As the private sector possesses several strengths that can aid in addressing the challenges associated with the rapid transformation of the health care system. (26,27). Additionally, due to the high prevalence of chronic diseases and unhealthy lifestyles in Saudi Arabia, we are in eager need to adopt lifestyle medicine practice that has a great impact on both health promotion and disease prevention (28).

The main limitations of this study are its cross-sectional design; the causal relationship could not be determined, and it limits the generalizability of our results. In addition, the potential impact of unmeasured confounders on the study results are other limitations. The main strength of our study was its being the first study, which shows the lifestyle factors and their association with QoL among patients with chronic diseases at the primary health care centers in Riyadh of Saudi Arabia, and its large sample size. Additionally, a valid and comprehensive Arabic questionnaire was used for determining the QoL, and the lifestyle factors included in the study questionnaire were adopted from based on the WHO STEP approach.

In conclusion, the current study showed high level (69.3%) of good total QoL, among patients with chronic diseases at the primary health care centers in Riyadh of Saudi Arabia. In addition, unhealthy lifestyle factors including obesity, poor cooking practices, and eating meals outside home were the main determinants that impaired the level of the QoL among the studied population. Periodic and regular monitoring of the QoL among patients with chronic diseases should be considered in the primary health care centers in Riyadh of Saudi Arabia. Additionally, interventions including educational components for supporting self-management could be implemented in primary health care settings to improve QoL among patients with chronic diseases. Further future studies are required to confirm these findings.
